# The role of endoscopic ultrasound on the preoperative T staging of gastric cancer: A retrospective study

**DOI:** 10.1097/MD.0000000000004580

**Published:** 2016-09-09

**Authors:** Chaoqun Han, Rong Lin, Huiying Shi, Jun Liu, Wei Qian, Zhen Ding, Xiaohua Hou

**Affiliations:** Division of Gastroenterology, Union Hospital, Tongji Medical College, Huazhong University of Science and Technology, Wuhan, China.

**Keywords:** accuracy, endoscopic ultrasonography, gastric cancer, staging

## Abstract

Endoscopic ultrasonography (EUS) is used for preoperative assessment of gastric cancer. However, recent studies suggested that EUS staging accuracy is lower than previously thought. We aimed to assess EUS efficacy and image characteristics in preoperative gastric cancer T staging.

A retrospective review of clinical and imaging features of 232 gastric carcinoma patients who underwent preoperative EUS assessment of T stage was performed. Only cases with tumor-free resection margin status and no metastases were enrolled. Comparisons of preoperative EUS and postoperative histopathological stagings were also performed to identify vital EUS image features for evaluating gastric carcinoma.

EUS accuracy for T staging was 64.2% (149/232) with the highest accuracy for T3 (75.0%). Enlarged lymph nodes, well differentiated histological type and Borrmann IV type were associated with diagnostic accuracy in predicting tumor invasion. Although no factors were associated with overstaging, circumferential lesions ≥1/2, signet ring cell adenocarcinoma, and Borrmann IV type had significantly higher risks of understaging. Gastric wall outer edge irregularity was also an indicator of serosal involvement with a sensitivity of 82.0%. The pancreas and colon were more frequent disease extension sites than previously predicted.

Although EUS is likely the best and most accurate option that we have used to stage gastric cancer, the finding that factors including circumferential lesions, signet ring cell adenocarcinoma, and Borrmann IV type carcinoma were more frequently related to incorrect staging warrants attention.

## 1 Introduction

Gastric cancer is a common malignancy that has a poor prognosis and high mortality.^[1,2]^ The most important method for evaluating prognosis is the staging of the cancer. Factors that are considered during staging include infiltration depth (T staging), lymph node (N staging), and distant organ metastasis (M staging). For example, the 5-year survival rates for stages T1, T2, T3, and T4 are 86.9%, 76.3%, 64.6%, and 31.1%, respectively.^[3]^ Moreover, since results from preoperative staging often direct therapy decisions, accurate staging is important for selecting the most effective treatments.

Endoscopic ultrasonography (EUS) provides detailed images and is routinely used to detect and stage gastrointestinal cancers.^[4,5]^ Many studies have focused on the role of EUS in preoperative staging of gastric cancer, and EUS is indeed often considered as the first-choice imaging modality for regional staging of gastric cancer compared to other methods.^[5–8]^

However, there are several contradictory reports about the accuracy of EUS for T staging of gastric cancer since reported values for EUS diagnostic accuracy in overall T staging varied from 42.6% to 87.7%.^[9–11]^ In our study, we evaluated the accuracy rate of EUS for gastric cancer staging. Furthermore, we attempted to identify factors that affect the accuracy of EUS staging.

## 2 Materials and methods

### 2.1 Patients

A total of 272 patients with gastric cancer presenting to the Department of Gastroenterology, Union Hospital, Wuhan, China over the 3-year study period (January 2012–January 2015) were included. Each diagnosis was pathologically confirmed using samples obtained through routine esophagogastroduodenoscopy (EGD) with biopsy. In addition, preoperative staging using EUS and postoperative pathological staging were both performed for all patients. To obtain correct histological staging, surgical samples were required to have a tumor-free resection margin status. We retrospectively collected data for 232 patients who were diagnosed with gastric cancer with confirmed pathological staging. Forty patients were excluded from the analyses due to distant metastases, lack of surgery, or having undergone preoperative chemoradiotherapy or palliative surgery. The study was approved by the institutional ethical review committee. Patients signed informed consent for EUS operation, and data had been anonymized and deidentified.

### 2.2 EUS equipment and technique procedures

EUS was performed using the Olympus processor EU-ME1 and F75 with a standard radial scanner (Olympus America, Inc., Center Valley, PA). The gastric lumen was filled with 300 to 800 mL of degassed water or fitted with a water-filled balloon to improve transmission of the ultrasound beam with variable frequencies of 5, 7.5, 12, and 20 MHz. The tumor infiltration depth was imaged as a hypoechoic disruption and evaluated based on the 5-layered gastric wall structure.^[12]^ Assessment of tumor invasion depth by EUS was made in accordance with the American Joint Committee on Cancer (AJCC) tumor, node, metastasis (TNM) Classification (sixth edition).^[13]^ The features of the different stages are as follows—T1: tumor invasion limited to the mucosal or submucosal layer, T2: destruction of the muscularis propria or subserosal layer, T3: cancer penetrating the serosa, and T4: disease invasion in the vicinity of the stomach or other organs. All operations were performed by 2 well trained (>1500 EUS procedures) endoscopists.

### 2.3 Data collection

To determine the value of EUS to evaluate T staging of gastric cancer lesions, we retrospectively collected factors that influenced accurate diagnosis of tumor invasion depth. Evaluated data included patient demographics (i.e., gender and age), clinicopathologic details (i.e., tumor location, histological type and Borrmann classification), or ultrasonic characteristics (i.e., circumferential spread). Diagnostic accuracy, overstaging, and understaging for preoperative EUS T staging were compared with each pathological finding according to AJCC guidelines. Tumor locations were categorized using 2 criteria. One group was divided into circumferential lesions ≥1/2 or circumferential lesions <1/2 based on the cross-sectional circumference, and the other group was divided according to lesion locations (fundus, corpus, gastric angle, and antrum) relative to the longitudinal axis of the stomach. The histologic types were in accordance with the World Health Organization classification^[14]^ and classified into well differentiated, moderately differentiated, or poorly differentiated tubular adenocarcinoma, and signet ring cell adenocarcinoma. The characteristics of Borrmann classification were as follows: type I, polypoid or fungating without ulceration; type II, ulcerating lesions surrounded by elevated borders; type III, ulcerating lesions with infiltration of the gastric wall; and type IV, diffusely infiltrating tumor without any craters or elevated lesions that is macroscopically widespread (linitis plastica). Because preoperative assessment of lymph node metastasis was also evaluated by computed tomography (CT), and EUS has a poor diagnostic success rate for N/M stage,^[9,15,16]^ the accuracy of EUS N/M staging and other related data is not shown.

### 2.4 Statistical analysis

Continuous variable results were presented as mean ± standard deviation. Associations among various categorical variables were analyzed by Pearson chi-squared test and noncategorical variables were evaluated by *t* tests. Subsequently, a binary or multivariate logistic regression analysis was constructed to explore the factors that affected EUS T staging accuracy. Statistical analyses were performed with SPSS software 19.0 (SPSS Inc., Chicago, IL). A *P* value <0.05 was defined as statistically significant.

## 3 Results

### 3.1 Demographic, histological, and endoscopic characteristics

This study evaluated 232 patients who met all defined criteria. There were 127 men and 105 women with a mean age of 54.76 years (range 27–86 years). Patients in the study group had tumors located in the fundus (24.1%), corpus (27.6%), gastric angle (9.9%), and antrum (38.4%). The numbers of well differentiated, moderately differentiated, poorly differentiated, and signet ring cell adenocarcinoma were 44 (19.0%), 42 (18.1%), 77 (33.2%), and 69 (29.7%), respectively. We also focused on EUS image characteristics, including presence of circumferential lesions (cancer extension beyond a semicircular area, 27.2%), local enlargement of lymph nodes (37.5%), and ascites (9.9%). The clinical and pathological features of the enrolled cases are shown in Table 1.

**Table 1:**
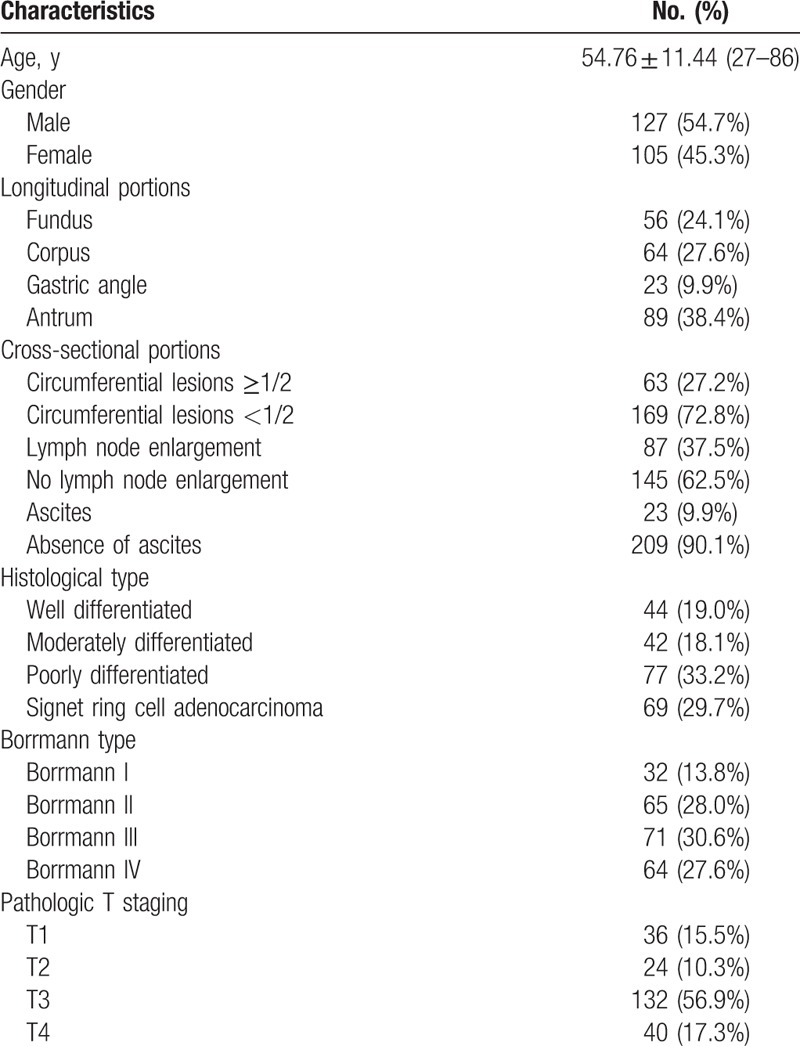
Basic tumor characteristics and pathological stage of 232 gastric cancer patients.

### 3.2 EUS T staging accuracy

The overall accuracy of EUS for T staging was 64.2% (149/232). Among the different stages, the accuracy was 33.3% for T1, 62.5% for T2, 75.0% for T3, and 57.5% for T4. Furthermore, the differences in accuracy rates for the 4 stages were statistically significant (*P* = 0.023). The overstaging and understaging rates were 12.9% (30/232) and 22.9% (53/232), respectively (Table 2).

**Table 2:**
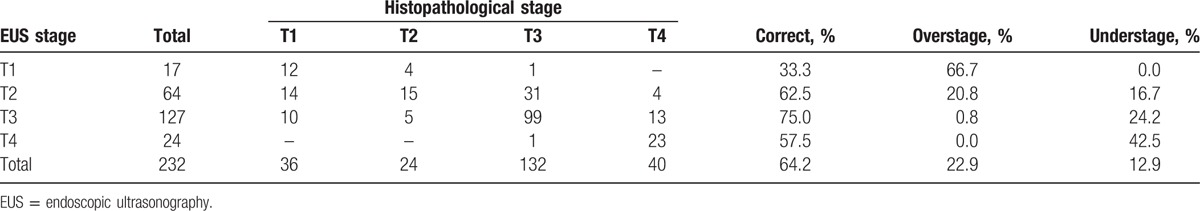
EUS and histopathologic results for T staging in 232 gastric cancer patients.

### 3.3 Factors influencing evaluation of EUS gastric cancer staging

**Figure 1:**
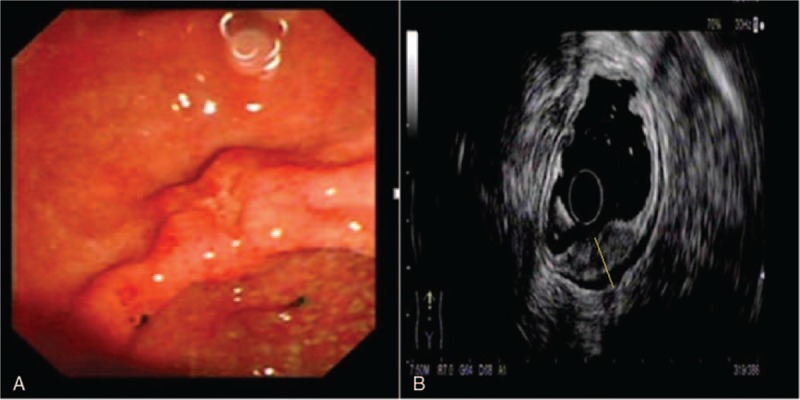
Correct diagnosis of T staging in a patient with well differentiated gastric cancer. (A) Endoscopic image of the gastric cancer showing an ulcer located in the anterior wall of the antrum; (B) EUS image of the lesion showing a 13-mm thick hypoechoic lesion spreading from the mucosal to muscularis propria layers with an intact serosa layer (dotted line). Surgical resection confirmed well differentiated gastric cancer infiltrated to the muscularis propria layer.

**Figure 2:**
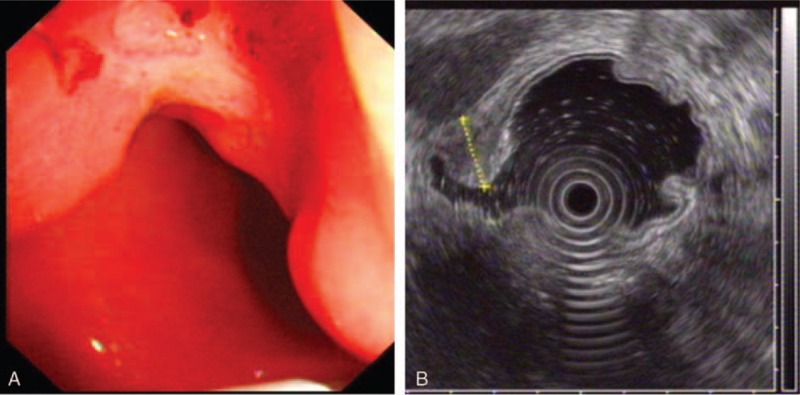
Incorrect diagnosis of T staging in a case of poorly differentiated and partial signet ring cell gastric cancer. (A) Endoscopic image of the lesion showing an ulcer lesion located in the gastric antrum; (B) EUS image of the lesion showing a 17-mm thick hypoechoic lesion that spreads throughout the entire wall and invades the serosa invasion (dotted line). The corresponding surgical specimen confirmed poorly differentiated and partial signet ring cell gastric cancer confined to the submucosal layer.

Among the patients enrolled in this study, EUS diagnostic accuracy was not influenced by the presence of ascites or cancer location (Table 3). However, multivariate analysis showed that the presence of circumferential lesions (cancer extension in more than a semicircle) presented a significant risk of incorrect staging (*P* = 0.048, odds ratio [OR] = 1.816; Table 4). In contrast, accurate staging of tumors was enhanced by the presence of local enlarged lymph nodes (*P* = 0.048). For well differentiated tumors or Borrmann I type cancer, EUS had better staging success relative to that for signet ring cell carcinoma (77.3% vs 49.3%, *P* = 0.001; Figs. 1 and 2) and Borrmann IV type (84.4% vs 46.9%, *P* = 0.000; Table 3). When subjected to multivariate analysis, lesions with signet ring cell adenocarcinoma also presented a significant risk factor for accuracy with a 2.574-fold OR (*P* = 0.024; Table 4).

**Table 3:**
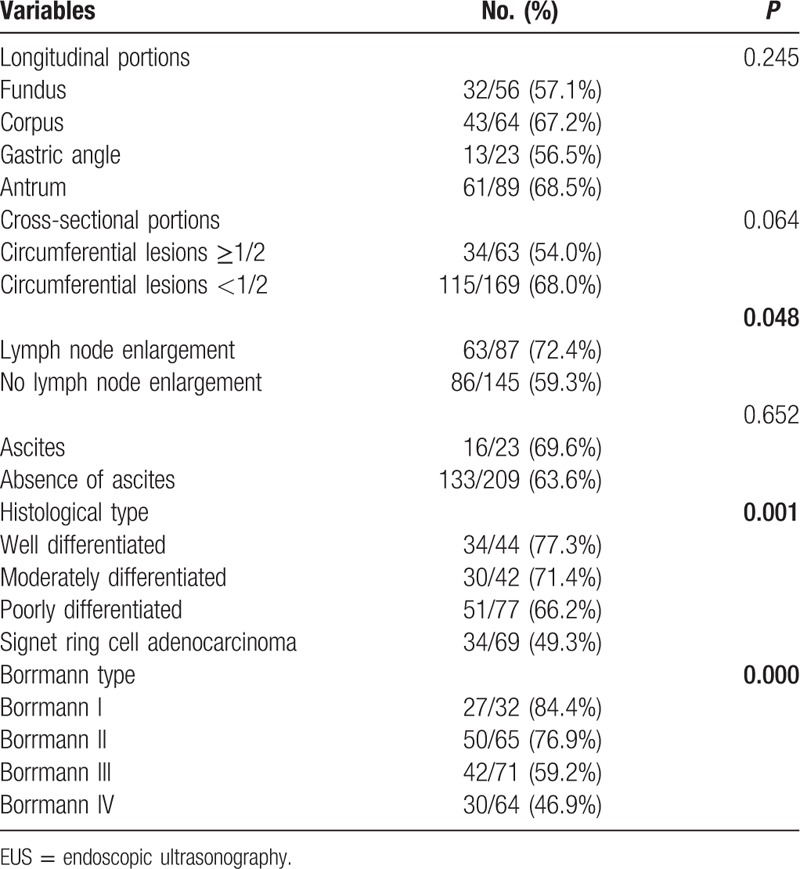
EUS T staging accuracy according to clinicopathologic and endoscopic variables.

**Table 4:**
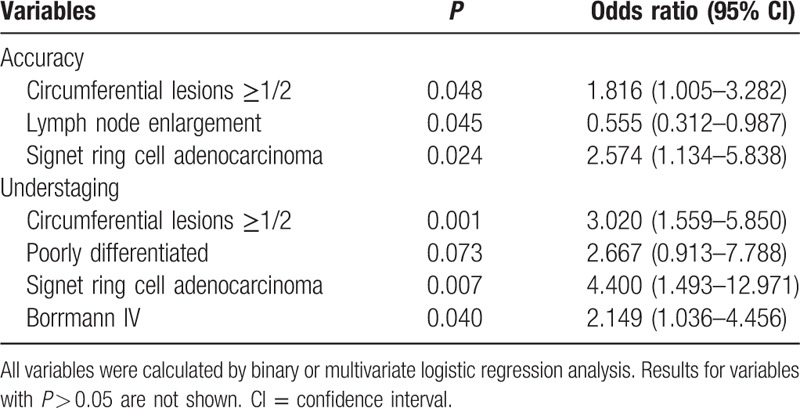
Multivariate analysis of clinicopathologic factors affecting EUS T staging.

### 3.4 Risk factors for overstaging and understaging

Circumferential lesions had a greater possibility of understaging (*P* = 0.001). Furthermore, signet ring cell carcinoma (*P* = 0.015) and Borrmann IV type gastric cancer (*P* = 0.000) also had a significantly higher risk of understaging (Table 5). When these factors were subjected to multivariate analysis, they remained significant (Table 4). However, no meaningful clinical features appeared to increase the risk of overstaging.

**Table 5:**
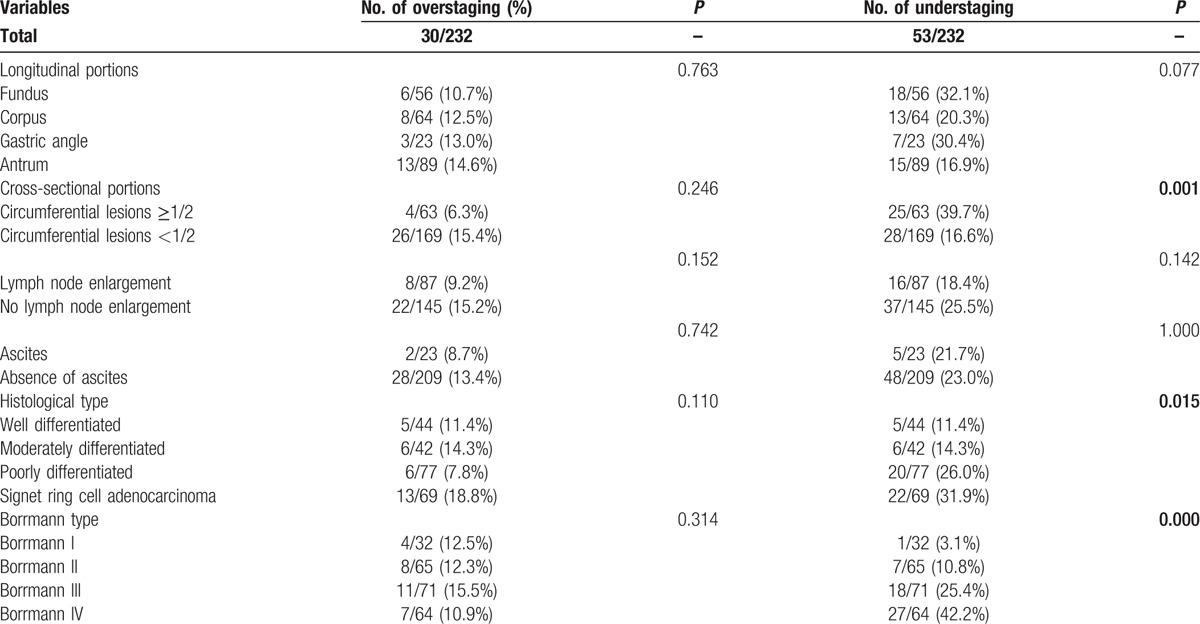
Factors affecting EUS over- or understaging.

### 3.5 Gastric wall outer edge irregularity is an effective indicator for confirming serosal involvement

In our study, irregularities in the outer edge of the gastric wall were a marker of gastric serosal layer involvement. The consistency rate between EUS and pathological results for serosal extension was 79.7% (185 of 232). Furthermore, sensitivity and specificity values, positive predictive values (PPVs), and negative predictive values for this characteristic were 82.0%, 73.3%, 89.8%, and 58.7%, respectively.

### 3.6 The incidence of adjacent organ involvement in gastric cancer

In this study, cancer extension to adjacent organs was confirmed by pathological results for 12 cases: 1 (8.3%) in liver, 1 (8.3%) in spleen, 5 (41.7%) in pancreas, and 5 (41.7%) in colon. However, only liver involvement was predicted in preoperative EUS examinations.

## 4 Discussion

Therapies for treating gastric cancer are often selected according to staging results. Therefore, accurate preoperative staging is important for selecting the most effective treatments.^[17–21]^ EUS is routinely used in preoperative staging of gastric cancer because different structural layers of the gastric wall show remarkable differences in their echogenic appearance. However, despite these differences, the accuracy of EUS for staging gastric cancer varied across several studies. The diagnostic accuracy of EUS for overall T staging varied from 56.9% to 87.7%, and the pooled accuracy of T1, T2, T3, and T4 stages were 14% to 100%, 24% to 90%, 50% to 100%, and 25% to 100%, respectively.^[22–24]^

In our study, the accuracy of T staging was only 64.2%, which is consistent with that reported in previous studies.^[25–27]^ Moreover, the accuracy of T1 stage predictions was lower than expected and had a high incidence of overstaging (66.7%), which may have resulted from local inflammation, edema, and fibrosis that in turn produced hypoechoic changes that made distinguishing tumors difficult.^[16,28,29]^ In our study, we found that 25% (9/36) of stage T1 gastric cancers presented with increased thickness of the muscularis propria that is often considered to be an indicator of stage T2. This feature is one cause of overstaging. Therefore, mild thickening of the muscularis propria layer may be due not only to cancer extension (T2 staging) but also inflammation (T1 staging).

In our study, T2 staging accuracy was 67.5%, which is also similar to previous reports.^[25,30–32]^ The challenge of accurate T2 stage still remains a frequent issue since this T stage is commonly overstaged.^[33]^ From a technical perspective, distinguishing between subserosal (T2b) and serosal (T3) lesions by EUS is challenging. Furthermore, some endoscopists prefer to assign a higher T stage when there is insufficient evidence to differentiate between 2 stages. Moreover, in the fundal and lesser curvature regions of the stomach, the gastric wall is not entirely covered by the serosa, which may further confound gastric cancer staging.^[34]^

The accuracy rate of T3 staging was the highest in preoperative staging (75%) and accounted for the most cases of understaging (24.2%). These results indicate that EUS is an accurate approach for evaluating serosal involvement (T3 and T4 cases), largely because irregularities in the serosal layer are good indicators of cancer involvement (PPV nearing 90%). Nonetheless, some disease extension to adjacent organs was missed because of the assumption that, relative to other organs, the liver is most frequently involved in gastric cancer. Indeed, some studies showed that the rate of liver metastasis in gastric cancer can reach 5% to 9%, although pancreas involvement is rare.^[35–38]^ Interestingly, we found that the pancreas and colon were more likely than previously thought to be involved in gastric cancer. Therefore, gastric cancer extension from the stomach to other organs, particularly the pancreas and colon, merits consideration.

Our results show that there were no factors aligned with overstaging, but circumferential lesions, signet ring cell adenocarcinoma, and Borrmann IV carcinoma were independent indicators that were associated with EUS understaging of gastric carcinoma. These results indicated that cancers with these characteristics may be more severe than that indicated by EUS preoperative staging and that cancer invasion could be occurring on a microscopic level that cannot be detected by EUS. Careful attention is required during EUS examination, which must precede treatment planning for gastric cancer with these features. Improvement in EUS techniques and equipment will be essential to overcome the weak points.

Accurate preoperative staging is greatly essential for proper stage-dependent patient management. EUS T stage has significant shortcomings; however, it is also likely the best and most accurate staging option that we have. Mocellin and Pasquali^[8]^ reported that the pooled sensitivity and specificity of EUS in identifying T1 to T2 (superficial) versus T3 to T4 (advanced) gastric cancer were 0.86 (95% confidence interval [CI]: 0.81–0.90) and 0.90 (95% CI: 0.87–0.93), respectively. The pooled sensitivity and specificity in discriminating T1 (early gastric cancer) versus T2 (muscle-infiltrating) tumors were 0.85 (95% CI: 0.78–0.91) and 0.90 (95% CI: 0.85–0.93), respectively. Even for the diagnostic capacity of metastatic lymph nodes involvement, the pooled sensitivity and specificity were 0.83 (95% CI: 0.79–0.87) and 0.67 (95% CI: 0.61–0.72), respectively. Certainly, the present study has its inherent limitations that should be considered. First, the study is retrospective, and the samples of patients are relatively small suggesting restricted application of the results; second, information on adjuvant chemotherapy was not available in our analyses; furthermore, T stage including a subgroup, such as T1a versus T1b or T2a versus T2b, should be paid further attention. Finally, the fact that the accuracy of EUS N/M staging not shown in this study is another limitation should be considered. Although preoperative CT assessment of lymph node metastasis was performed for patients in this study, EUS is also a reliable method for assessing metastasis to lymph nodes that are adjacent to the stomach.^[8]^ A multicenter prospective study with a larger patient cohort that includes accuracy of EUS for detailed TNM stage is needed.

In conclusion, EUS can serve as an accurate method to determine the invasion depth of gastric cancer, although some overstaging and understaging can occur. Gastric cancers with circumferential lesions, signet ring cell adenocarcinoma, or Borrmann IV type were more frequently associated with incorrect staging and could predict the discordance of EUS with histological findings. For patients with these tumors, surgeons should consider the effect these features may have on staging and select treatment modalities accordingly.
